# Ultra-thin and smooth transparent electrode for flexible and leakage-free organic light-emitting diodes

**DOI:** 10.1038/srep09464

**Published:** 2015-03-31

**Authors:** Ki-Hun Ok, Jiwan Kim, So-Ra Park, Youngmin Kim, Chan-Jae Lee, Sung-Jei Hong, Min-Gi Kwak, Namsu Kim, Chul Jong Han, Jong-Woong Kim

**Affiliations:** 1 Display Components & Materials Research Center, Korea Electronics Technology Institute, Seongnam 463–816, Korea; 2 Display Convergence Research Center, Korea Electronics Technology Institute, Seongnam 463–816, Korea; 3 Department of Mechanical Design and Production Engineering, School of Engineering, Konkuk University, Seoul 143-701, Korea

## Abstract

A smooth, ultra-flexible, and transparent electrode was developed from silver nanowires (AgNWs) embedded in a colorless polyimide (cPI) by utilizing an inverted film-processing method. The resulting AgNW-cPI composite electrode had a transparency of >80%, a low sheet resistance of 8 Ω/□, and ultra-smooth surfaces comparable to glass. Leveraging the robust mechanical properties and flexibility of cPI, the thickness of the composite film was reduced to less than 10 μm, which is conducive to extreme flexibility. This film exhibited mechanical durability, for both outward and inward bending tests, up to a bending radius of 30 μm, while maintaining its electrical performance under cyclic bending (bending radius: 500 μm) for 100,000 iterations. Phosphorescent, blue organic light-emitting diodes (OLEDs) were fabricated using these composites as bottom electrodes (anodes). Hole-injection was poor, because AgNWs were largely buried beneath the composite's surface. Thus, we used a simple plasma treatment to remove the thin cPI layer overlaying the nanowires without introducing other conductive materials. As a result, we were able to finely control the flexible OLEDs' electroluminescent properties using the enlarged conductive pathways. The fabricated flexible devices showed only slight performance reductions of <3% even after repeated foldings with a 30 μm bending radius.

Organic light-emitting diodes (OLEDs) have received much attention as thin and flexible electronic devices^1^,^2^,^3^,^4^,^5^,^6^,^7^,^8^,^9^,^10^. In general, OLEDs have been fabricated by the sequential deposition of thin organic layers and a metallic layer on a patterned indium tin oxide (ITO) anode. In this process, leakage current can arise from the spikes on the ITO anode and the short path between the anode and cathode^11^,^12^. Previous studies have demonstrated that such a leakage current degrades the stability and efficiency of OLEDs^13^,^14^,^15^. One of these studies^13^ also revealed that a simple plasma irradiation on the anode alleviated the leakage current by planarizing the surface of the anode. However, a series of additional processes such as a photolithography and sputtering are required to prevent a short due to the proximity of the two electrodes. In this process, a thick insulating layer formed on the anode separates the anode from the cathode. In this case, the leakage current can be lowered in exchange for increased thickness and cost of the OLED device.

Several studies on the fabrication of flexible transparent conductors have provided another valuable hint toward resolving this surface-purity issue by employing a silver nanowire (AgNW)-polymer composite^16^,^17^,^18^,^19^. These works proposed an entirely new approach: coating a liquid polymer onto pre-coated AgNW networks then drying and peeling off the resulting composite from the supporting substrate, which yields a solid and smooth conductive surface by burying the AgNW film in the surface of the polymer matrix. It appears that the issues with rough surfaces and ITO edges projecting out from the substrate may be resolved by such an approach. However, since the AgNWs are buried inside the polymer matrix, only a very small area is exposed at the surface. This results in a very limited conductive pathway. Consequently, the charge injection into the active layer of such an OLED is poor. To address this matter, Li et al. suggested that a pre-coat of carbon nanotubes (CNTs) (before coating the AgNW dispersion) could promote conductive coverage at the surface of the composite electrode^20^. However, the use of additional material (CNT) increases the fabrication cost and worsens the transmittance of the films. One more point worth noting is that most previous studies employed poly(3, 4-ethylenedioxythiophene):poly(styrenesulfonate) (PEDOT:PSS) to further reduce surface roughness. PEDOT:PSS is a popular material for enhancing charge injection and surface planarization, but it is not employed in the fabrication of today's dry-processed OLEDs because of its wet-based processing. It should also be noted that the acidity of PEDOT:PSS may lead to the corrosion of AgNWs, especially when it is exposed to a high-temperature, humid environment. An example of this is shown in Fig. S1. Several additional issues should also be addressed regarding the careful design of a device structure, such as a large decrease of optical transmittance by a thick overcoating of PEDOT:PSS on AgNWs (as shown in Fig. S2) and its poor color neutrality. Therefore, it is necessary to develop an ultra-smooth electrode with high conductive coverage on the surface, without employing additional conductive materials. Studies on composite-based electrodes using more than a single material are provided elsewhere^21^,^22^,^23^,^24^,^25^,^26^,^27^,^28^.

Here, we used a colorless polyimide (cPI) to make an embedded-AgNW composite electrode, and hence, a smooth and flexible transparent anode for use in OLEDs. The cPI employed in this study was highly transparent and colorless, while exhibiting superior mechanical properties (a Young's modulus of 6 GPa and a tensile strength of 300 MPa). Therefore, the composite film could be fabricated with a thickness of less than 10 μm, which is advantageous in fabricating ultra-flexible substrates. Furthermore, such a film can withstand high-intensity irradiation and absorb less than 1% of water, making it ideal for use in flexible OLEDs. Practically, cPI is considered an industrially viable transparent substrate for flexible devices. To fabricate our composite electrodes for small-molecule OLEDs without additional planarization materials, we introduced a sacrificial layer for an improved peeling-off procedure. Furthermore, a simple and selective plasma etching was used to enlarge the surface coverage of the buried AgNWs without severe deterioration of the composite surface's smoothness. By this approach, an improved smoothness (comparable to sputtered ITO film or glass) and a high flexibility (resisting damage for a bending radius of up ~30 um) or even folding without large change of resistance was achieved. Furthermore, sky-blue, phosphorescent OLEDs were successfully fabricated on the composite electrode, exhibiting a leakage-free and foldable character.

## Results and Discussion

**Figure 1:**
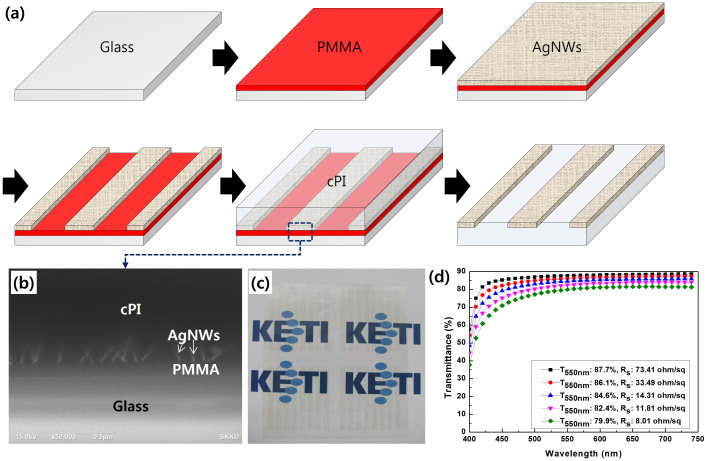
Fabrication of transparent and conductive films having extreme flexibility and smoothness: (a) a schematic description for the fabrication procedure, (b) a cross-sectional view of the Ag-nanowire-embedded structure processed by a FIB, (c) a fabricated AgNW-cPI composite film on printed Korea Electronics Technology Institute (KETI) emblems, (d) measured transmittance of the fabricated transparent electrode with varying AgNW density (including the transmittance of cPI).

The fabrication procedure for the AgNW-cPI composite is schematically illustrated in Fig. 1(a). First, a sacrificial layer—to be removed by a specific method, e.g., dissolution by an organic solvent or decomposition by exposure to ultra violet (UV) or laser light—is coated on a pre-cleaned glass substrate. Here, we employed poly(methyl methacrylate) (PMMA) as a sacrificial layer, which is easily dissolved by acetone for delamination of the AgNW-cPI composite film from the supporting glass. A PMMA suspension in chlorobenzene was coated on a glass sheet by a spin casting, followed by a baking at 120°C. An AgNW dispersion in isopropyl alcohol (IPA) was deposited on the surface of the PMMA to form a transparent and conductive network. After drying and patterning the AgNW networks, a cPI varnish was spin-coated onto the AgNW network and subsequently cured at 150°C for one hour. The thickness of the composite films was precisely controlled by varying the spinning velocity, and we fabricated samples with thicknesses of 7–8 μm, which is a suitable value for both extreme flexibility and reliable performance. In fact, the thickness could most likely be reduced to less than 5 μm without any loss of mechanical stability. Figure 1(b) shows a cross-sectional scanning electron microscope (SEM) image for the AgNW-cPI composite fabricated on glass. The buried AgNWs on the surface of the cPI, as well as the sacrificial PMMA layer, are clearly seen in this figure. Then, the composite films were peeled off from the glass by immersing the samples in acetone for 2 min. When the composite films were directly delaminated at ambient conditions without the introduction of a sacrificial layer, a rough surface with some AgNWs extruding from the originally buried sites was observed (see Fig. S3). It is worth noting that the roughness measured from peak to valley (R_pv_) is large enough to cause a vertical short or leakage, while the root mean square (RMS) value would indicate stability. This implies that the surface state of an electrode cannot be determined based solely on a single parameter of roughness (either an R_pv_ or R_RMS_ value). After drying at room temperature for 5 min, highly transparent and conductive films with line patterns were obtained, as shown in Fig. 1(c). As can be seen in the figure, the fabricated film is very clear and nearly haze-free.

The transmittance of the fabricated composite films with various AgNW densities is presented in terms of the corresponding sheet resistance in Fig. 1(d). The transmittance was measured with a UV–vis spectrometer using air as a reference (see Fig. S4 for the spectrum excluding cPI). A simple Meyer bar coating was employed to deposit the AgNW networks, and the coating density was controlled by varying the diameter of the steel wire rolled over the rod. As the AgNW density increased, the curve for the transmission spectrum moved downward with a steady decrease in average sheet resistance. With a maximal increase of single-coating density, we achieved a sheet resistance of less than 10 Ω/□, which rivals the performance of sputtered and thermally annealed ITO-on-glass substrates for organic devices. For further decrease of sheet resistance, a multiple-coating procedure is needed, otherwise several Ag spots are observed, due to a severe aggregation of the nanowires.

A figure of merit (FoM), defined as σ_DC_/σ_Op_ where σ_DC_ is the direct current conductivity and σ_Op_ is the optical conductivity of the composite film, was calculated from the measured transmittance and corresponding sheet resistance^29^,^30^. The FoM of the on-glass AgNW networks was 383, which increased to 426 after embedding them into the surface of the cPI. Such an increase to the FoM is mainly attributed to the decrease in contact resistance among the nanowires by a shrinkage of cPI during the baking and a perfect (lossless) transfer of AgNWs from the supporting substrate to the cPI matrix. This value is much higher than that of ITO film (FoM ≒ 100) and other competing materials for flexible transparent electrodes such as carbon nanotubes, graphene, and PEDOT:PSS (ranging from 30 to 120)^30^. The transmission spectrum of the AgNWs buried in the surface of the cPI is flat over the visible range for films of various densities. This trait is relevant from a technological perspective, since it leads to a neutral coloration, which is favorable for most applications. In comparison to that of a sputtered ITO, the spectrum is more stable, especially between 400 and 500 nm, which indicates that AgNW-cPI composites may be ideal for the fabrication of blue luminous devices, rather than ITO films^31^.

**Figure 2:**
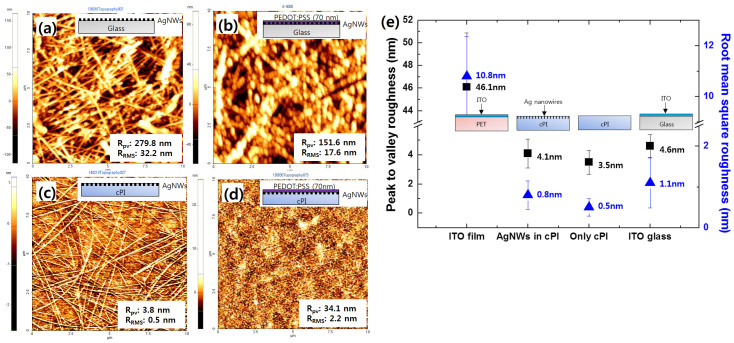
Comparison of morphologies by AFM analyses: (a) Ag nanowires coated on glass, (b) PEDOT:PSS coated on the sample from (a), (c) AgNW-cPI composite electrode, (d) PEDOT:PSS coated on the sample from (c), (e) a diagram comparing the roughness of various samples.

It is known that the high aspect ratio of AgNWs leads to a highly porous surface morphology, which is not advantageous for thin-film organic devices, where very smooth surfaces are required. We compared the morphology of various samples, e.g., AgNWs coated on glass, PEDOT:PSS/AgNWs on glass, a AgNW-cPI composite film, and a PEDOT:PSS/AgNW-cPI composite film, as shown in Fig. 2(a)–(d). One of the most straightforward strategies for smoothing porous AgNW surfaces is by coating with polymer material. However, as can be seen in the first two figures, the overcoating approach is not particularly efficient in planarizing the surface for preventing vertical shortage of the fabricated thin-film devices. Certainly, the surface state would be improved by a thicker coating of the PEDOT:PSS, however, the optical properties would then suffer from low transmittance (see Fig. S2) and its intrinsic bluish color. In contrast, the surface topography of the AgNW-cPI composite is extremely smooth (see Fig. 2(c) and (d)). An additional coating of PEDOT:PSS on the AgNW-cPI composite even caused a deterioration of the smoothness due to the partial agglomeration of the PEDOT:PSS particles. The R_pv_ and R_RMS_ roughness of the four cases are summarized in Fig. 2(e): ITO film, AgNW-cPI composite, plain cPI film, and ITO glass. It can be deduced that the high smoothness of the composite film originates from both the smoothness of the sacrificial PMMA surface, which was in contact with cPI, and the smooth release of the composite film from the supporting glass by the dissolution of the PMMA. More importantly, because the cPI varnish diffused and completely infiltrated the nanopores formed between the AgNWs and the PMMA, nanoholes or steps were scarcely present. The R_pv_ of the composite film is 4.1 nm, which is an order of magnitude lower than that of the ITO film (46.1 nm). Moreover, the R_pv_ and R_RMS_ of the composite electrode are on the same order as the bare cPI film and the ITO glass, which means that surface smoothness is dramatically improved by this approach.

**Figure 3:**
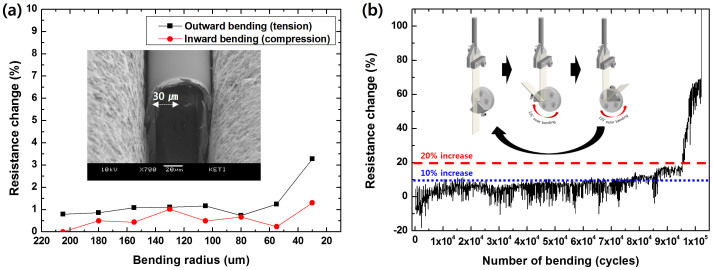
Mechanical stability of the composite film under different bending sequences: (a) change of the electrical resistance with varying bending radius (resistance was measured before and after bending), (b) change of the electrical resistance with number of bending cycles (bending radius: 500 μm).

To evaluate the AgNW-cPI composite electrode under compressive and tensile stress, two different testing methods were employed. We first measured the resistance change of samples being bent under radius of 30 μm to 200 μm, as shown in Fig. 3(a). The increased resistance during bending was restored to the initial value in all cases. The testing scheme is illustrated in Fig. S5 and S6. The measured resistance exhibited negligible change regardless of the compressive or tensile stress induced within the composite surface in the testing range, which demonstrates the high tolerance of the composite to bending stress. Owing to the low thickness of the fabricated composite, strain was restricted to 13% for outward bending (tensile stress) with a 30 μm bending radius. Nevertheless, to the best of our knowledge, this durable bending curvature is the smallest amongst any reported values. For evaluation of long-term reliability under a cyclic bending environment, equipment for automatic bending testing was employed. This apparatus enabled the electrode to experience alternate outward bending (tensile stress) and inward bending (compressive stress) repeatedly, resulting in cyclic fatigue failure (see the inset in Fig. 3(b)). This test used a bending radius of 0.5 mm, producing about 2% tensile and compressive strains. The electrode was bent at a cycle rate of 1 Hz and resistance measurement was recorded twice per cycle under inward and outward conditions. Figure 3(b) shows the percentage change of resistance (ΔΩ/Ω_0_) as a function of the number of bending cycles. The symbols ΔΩ and Ω_0_ are the actual change in resistance and initial resistance, respectively. These results show that the embedded electrode can maintain its electrical performance under bending for up to about 100,000 cycles. Alternate inward and outward bending leaves resistance virtually unchanged until around the 90,000th cycle. After this point, the resistance increases gradually, and then increases sharply after the 95,000th cycle. This exceptionally high mechanical stability is attributed to several factors. First, the ultra-low thickness (7–8 μm) of the cPI film results in a relatively small strain occurring at the surface of the composite electrode. Even if the sample was bent to a radius of 30 μm, only 13% of the strain is induced at the surface. Secondly, the strong mechanical properties of the cPI made it possible to resist degradation until after ~100,000 bending cycles at a bending radius of 500 μm, which might be the first example of such a durability in a commercially viable product, to the best of our knowledge. Third, good adhesion between AgNWs and cPI prevented the sliding of the wires at the interface (see Fig. S9). Lastly, and most importantly, the cPI varnish coating the AgNW networks fully permeated the AgNWs, filling the nanopores and gaps between each nanowire and the pre-coated PMMA on glass. This full coverage of the cPI made the composite extremely stable, even through folding sequences, although the surface coverage of the AgNWs was highly restricted. This is left for further discussion in terms of the emission properties of the fabricated OLEDs.

**Figure 4:**
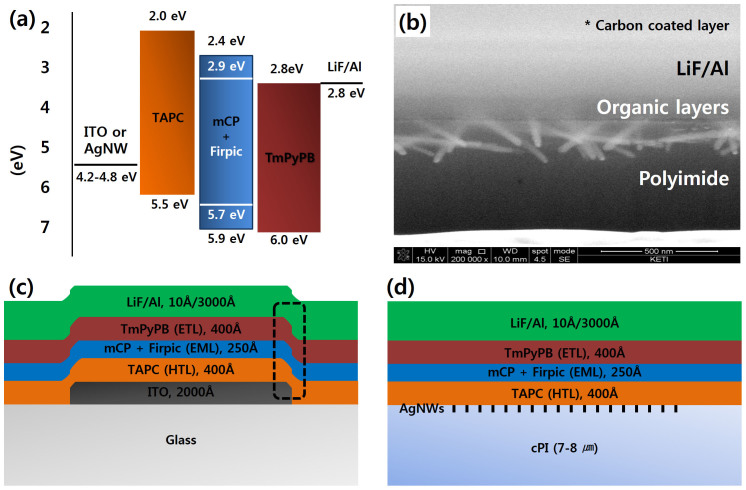
(a) A schematic diagram for energy levels and work functions of the chemicals and electrodes employed in this study, (b) a cross-sectional view of the fabricated device processed by a FIB, (c) a schematic description of the OLED device structure fabricated on a patterned ITO glass, and (d) a schematic description of the OLED device fabricated on the AgNW-cPI composite electrode.

Next, we fabricated a phosphorescent blue OLED device with the AgNW-cPI composite as a bottom electrode (anode). Because of the thermal stability of the cPI, the buckling instability induced by thermal deposition of the organic and Al layers was not a concern^32^,^33^. The OLED consisted of AgNW-cPI composite/TAPC/mCP(8 wt% Firpic)/TmPyBP/LiF/Al, as shown in Fig. 4(a). A cross-sectional image of the fabricated device is shown in Fig. 4(b). It appears that the height of the AgNW coverage (175 nm) is much larger than that of the organic layers (105 nm). From this, it could be deduced that the sole use of AgNWs is not a proper means for making bottom electrodes for OLEDs. For comparison, we fabricated two types of blue OLEDs, namely, an ITO-glass-based device shown in Fig. 4(c) and an AgNW-cPI-composite-based device shown in Fig. 4(d). The physical form of the two structures is completely different, that is, no edge effects are expected in the latter case.

**Figure 5:**
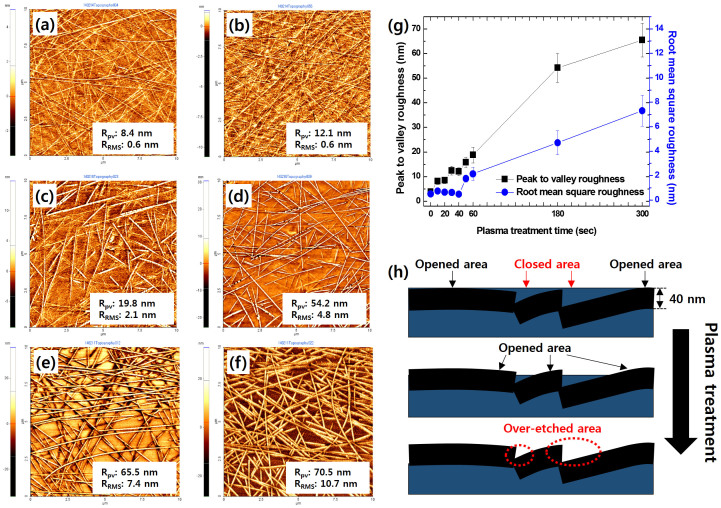
Effects of plasma treatment on the surface of AgNW-cPI composites: morphologies analyzed by AFM with treatment times of (a) 20 sec, (b) 40 sec, (c) 60 sec, (d) 180 sec, (e) 300 sec, and (f) 420 sec. (g) A graph of measured roughness with plasma treatment time, and (h) schematic descriptions for plasma treatment on the surfaces of the AgNW-cPI composites. Fully buried AgNWs are excavated by a simple plasma treatment.

**Figure 6:**
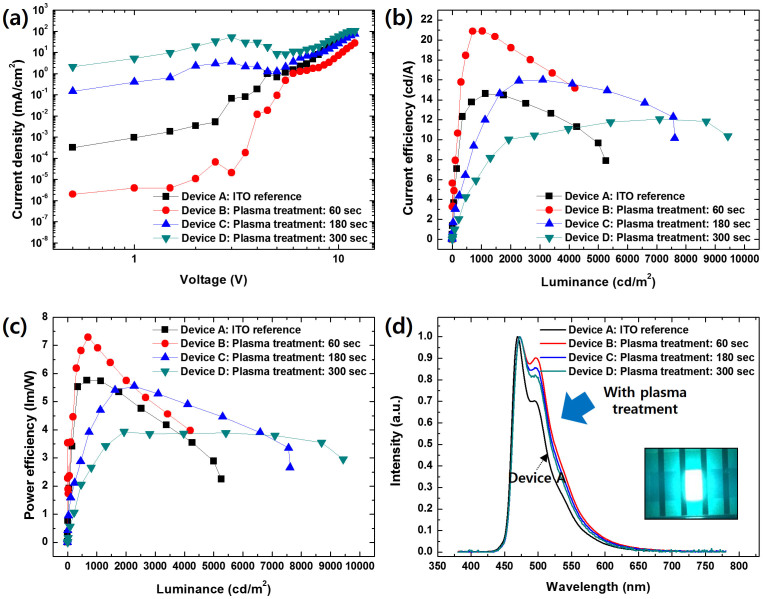
Characterization of the fabricated OLED devices: (a) current density–bias voltage, (b) current efficiency–luminance, (c) power efficiency–luminance, (d) electroluminescent intensity–wavelength.

Unfortunately, the turn-on voltage of the device was prohibitively high because of the low coverage of conductors and thin layers of cPI coating the buried AgNWs in the surface of the composite. In order to overcome this, we developed a selective etching method to remove the cPI layers covering the AgNWs, which enlarged the coverage of the conducting areas. Here, we employed plasma etching with Ar gases on the basis of two main factors. First, the etching rate could be precisely controlled by input power, flow rate of the gases, and etching time. For maintaining the smoothness of the composite electrode, accurate control of the etching rate is vital. Second, a selective etching was possible by means of the controllable input power. In order to improve device performance, we optimized the conditions for plasma treatment to remove the thin cPI layer without the formation of silver oxide. As the plasma exposure time was extended, the AgNWs that would have otherwise been buried became exposed with a concomitant increase in the surface roughness of the composite, as shown in Fig. 5. As mentioned earlier, these excavated AgNWs were expected to enhance the hole-injection properties of OLED devices. For plasma exposure times ranging between 10 s and 300 s, the R_RMS_ of the surface was less than 10 nm, which is suitable for an OLED's bottom electrode. Device A was fabricated as a reference, using an ITO electrode. The AgNW-cPI composites treated with plasma over the course of 60 s, 180 s, and 300 s were used for fabricating devices B, C, and D, respectively. As shown in Fig. 6, the J-V-L characteristics of the devices were measured until the voltage reached 12.0 V. Interestingly, the current density–voltage curves showed that the leakage current of device B (0.03 μA/cm^2^) was lower than that of device A (2.5 μA/cm^2^) by two orders of magnitude at a reverse bias voltage of 2.0 V. The higher leakage current for device A was attributed to the proximity between the anode and a cathode, as illustrated in Fig. 4(c). Since the current was concentrated on the perimeter of the anode, the leakage was severe without the insulting layer^34^. The leakage current of device B was comparable to that of the OLED fabricated with a 500 nm thick polyimide as an insulating layer on the ITO anode (0.034 μA/cm^2^)^35^. Despite the low leakage current of device B, the current density was lower in device B than device A because the holes were injected through sparsely dispersed AgNWs. However, the local current in the vicinity of the AgNWs lowered the turn-on voltage (4.5 V), which was comparable to that of device A. The current efficiency of device B was 41% higher than device A at a brightness of 1000 cd/m^2^, indicating that the local current and the low leakage current were advantageous in the AgNW electrode. The difference in their power efficiencies was rather small, thanks to the high operating voltage in device B. The brightness of device B exceeded 4000 cd/m^2^ at 12 V, suggesting that the AgNW electrode could be applicable for OLED luminaires. An efficiency roll-off was observed when the current densities of devices B and A surpassed 3.34 mA/cm^2^ and 7.79 mA/cm^2^, respectively. Presumably, the local current operated at the local emitting layer generated the narrow recombination zone in device B. As a result, this facilitated triplet–triplet annihilation in device B.

For devices C and D, a high leakage current induced by the high R_pv_ of the anode surface was observed. It was revealed that the R_pv_ of the surface was dramatically increased by irradiating plasma onto the AgNW-cPI composite, despite the small change in R_RMS_. The R_pv_ values of the AgNW-cPI for devices C and D were 54 nm and 65 nm, respectively. This also led to an increase in current density by enlarging the active surface area of the AgNWs, which contacted the hole-transporting layer (HTL) of the devices. The turn-on voltage of devices C (4.3 V) and D (4.1 V) were lower than that of device B, thanks to the expanded surface coverage of the AgNWs^20^. In addition, the roll-off efficiency was as follows: device B > device C > device D. The low roll-off was attributed to the balance between charges improving the hole-injection properties of devices C and D^36^. The roll-off was also related to the shift of the recombination zone in the OLED devices^37^. For device B, the recombination zone was located near the HTL/emission layer (EML) interface due to the imbalance between holes and electrons. The charge imbalance was dependent on the difference in the charge-injection barriers^38^,^39^. While the energy barrier for hole injection was ~1.0 eV, that for electron injection was negligible. Considering the charge trapping due to Firpic, the electron-injection barrier may be as low as 0.1 eV. This low barrier shifted the recombination zone toward the HTL/EML interface where triplet–polaron quenching was severe^36^. The increase in the hole-injection efficiency of devices C and D shifted the recombination zone far from the HTL/EML interface. The shift of the electron–hole recombination zone was confirmed by the presence of a hypsochromic shift in the emission spectrum as shown in Fig. 6(d). The microcavity effect then explains how the generated light was blue-shifted by reducing the intensity of the peak at 500 nm as the recombination zone moved toward the cathode^40^. The intensity at 500 nm was measured to show the decreasing order of device B > device C > device D. The Commission Internationale de'Eclairage (CIE) color coordinates were (0.168, 0.369) for device B and (0.166, 0.365) for device D. From this result, it may be concluded that the emission properties of an OLED based on AgNW-polymer composite could be delicately controlled by varying the duration of a simple surface treatment using plasma irradiation. The performance of the device could then likely be enhanced by optimizing the diode architecture.

**Figure 7:**
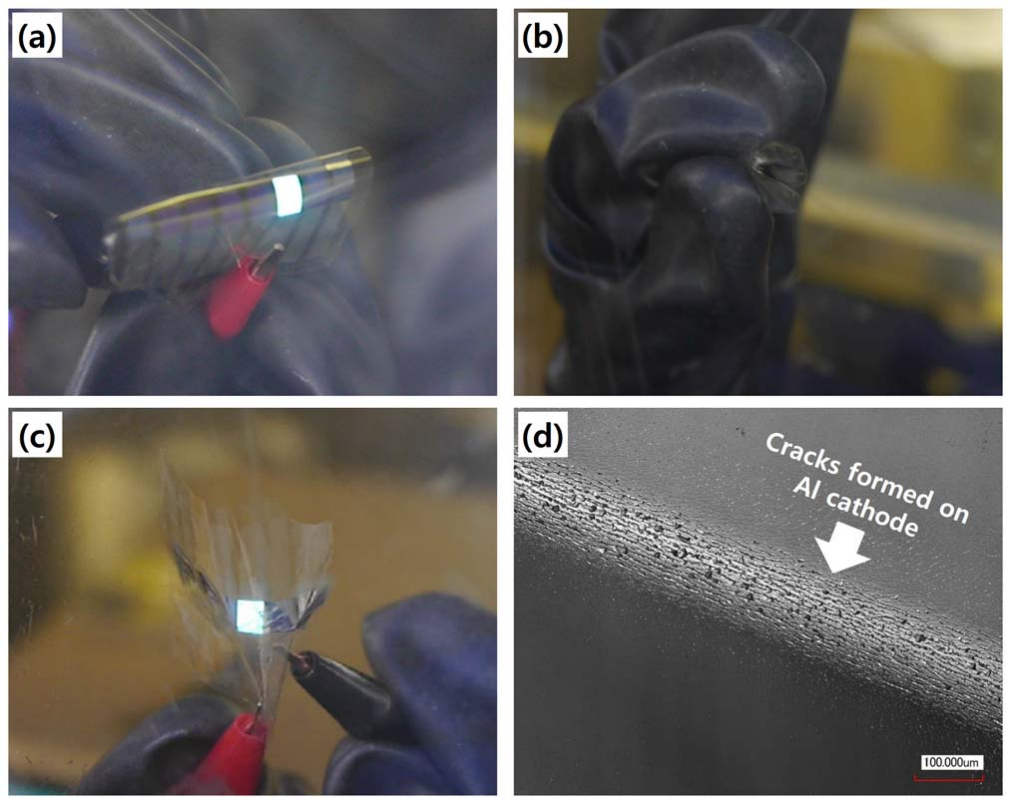
Bending stability of the flexible OLEDs based on AgNW-cPI composite electrodes: (a) a bent, lit OLED, (b) a crushed OLED, (c) an unfolded, lit OLED, and (d) cracks formed on an Al cathode after being repeatedly bent with a radius of 30 μm (100 times).

Given that the primary goal of using a flexible electrode in organic devices is to achieve highly reliable performance under conditions of continuous mechanical deformation, mechanical stability tests were carried out on the OLED devices fabricated as part of this study. Like the preceding tests with bare AgNW-cPI composite electrodes, the flexible OLEDs (fabricated with the optimum processing conditions and 120 s of plasma irradiation) were subjected to bending tests at a radius of 30 μm. After ten repetitions, the electroluminescence properties were not severely altered: average luminance decreased by only 2.1%. As shown in Fig. 7(a), (b) and (c), the fabricated flexible OLEDs endured various bending or crushing motions (see also the supporting movies). At some points, dark spots were observed, mainly attributed to the formation of cracks in the Al cathode, as shown in Fig. 7(d). Thus, the reliability could likely be drastically improved by replacing the Al cathode with more flexible materials or structures.

In summary, an ultra-thin and smooth AgNW-cPI transparent electrode has been prepared for use in foldable OLEDs by utilizing a solution-based AgNW embedding process. For producing ultra-thin films, a cPI with rugged mechanical properties was used. A sacrificial layer was employed to permit the smooth release of the composite electrode from glass, which left the surface of the thin composite ultra-smooth. From this, the extreme flexibility (resisting up to a 30 μm bending radius or even folding) was achieved without severe resistance increase, while the smoothness of the composite surface was comparable to that of glass. However, the surface coverage of conductive pathways (AgNWs) in these untreated composites is highly restricted, because most of the AgNW network is buried underneath the surface. To address this, we employed a simple plasma treatment for removing the thin cPI layer overlying the buried AgNWs without the formation of silver oxide. By this approach, the conductive coverage was easily enlarged, and thus the hole-injection properties were dramatically improved without introducing any other conductive materials. The OLEDs fabricated from such a composite electrode were extremely flexible, showing a stable performance with a luminance reduction of <3% after ten repeated bending at a radius of 30 μm. The developed process for the flexible electrode is easily scalable and highly reliable, and thus AgNW-cPI is believed to be a potential replacement for conventional sputtered transparent-metal-oxide materials.

## Methods

The procedure used for the fabrication of the AgNW-cPI transparent electrode is schematically illustrated in Fig. 1(a). First, a glass substrate is cleaned using detergent, de-ionized water, acetone, and isopropanol. The PMMA suspension in chlorobenzene was coated onto glass by a spin casting, followed by baking at 120°C. The resulting PMMA layer was designed to be 100–200 nm thick. The PMMA coated glass was placed on a Mayer-rod apparatus and several drops (0.5 ml) of a nanowire solution (Nanopyxis Ltd., Korea) were deposited onto it. The average diameter and length of the nanowires were around 35 nm and 30 μm, respectively (aspect ratio: 950–1000). Immediately afterwards, a Mayer rod #8 (R.D. Specialties, Inc., USA) was rolled over the drops to evenly spread the nanowire solution over the PMMA surface, which was then carefully dried under infrared illumination for 10 min. The AgNW layer was patterned by a diluted acidic solution (Chromium Etchant, Sigma-Aldrich). A colorless polyimide (cPI) varnish (Kolon, Korea) was then spin-coated onto the electrode and subsequently cured at 150°C for 1 hour. The thickness of the film was precisely controlled by varying the spinning velocity, and so a thickness of 7–8 μm was selected to provide a suitable balance of flexibility and reliability. Once the film was formed on the composite electrode, the sample was soaked in acetone for 2 min to dissolve the added PMMA layer, so as to allow the film to safely peel off from the supporting glass.

Scanning electron microscopy (SEM, JEOL Ltd., JSM6700F, Japan) was used to investigate the microstructure of the AgNW networks. Their optical transmittance was measured using a UV–visible spectrophotometer (Jasco, V-560, Japan). The sheet resistance was measured by a non-contact measurement system (Napson Corporation, EC-80P, Japan). The surface roughness was measured by atomic force microscopy (AFM, Park Systems, XE-100TM, USA), and the cross-section of each sample was prepared by a focused ion beam (FIB, JEOL Ltd., JIB-4601F, Japan) system. The mechanical stability of the AgNW-cPI film, after being peeled off from the glass support, was evaluated by two different methods. The first method evaluated the actual foldability of the films with small bending radii (from 210 to 30 μm). The sheet resistance was measured before and after folding, serving as an indicator for folding endurance of the composite electrode. An automatic bend-testing machine (Toyoseiki Ltd., MIT-DA, Japan) was then used to measure the long-term reliability under repeated bending cycles. A bending radius of 0.5 mm was used to induce a ~2% tensile and compressive strain. The electrodes were bent at a cycle rate of 1 Hz, and their resistance was measured during both the inward and outward bending cycles.

The fabricated AgNW-cPI composite electrode was heated at 50°C for 1 hour in a vacuum oven to remove any residual water, after which all of the organic and inorganic layers required to fabricate the OLED were deposited by thermal evaporation. The organic layers consisted of: (1) 1, 1-bis-(di-4-polyaminophenyl)cyclohexane as a HTL, (2) N,N-dicarbazolyl-3, 5-benzene (mCP) as a host material and bis[2-(4, 6-difluorophenyl)pyridinato-N,C2'] iridium picolinate (Firpic) as a dopant material (emissive layer: EML), (3) 1, 3, 5-tri[(3-pyridyl)-phen-3-yl]benzene (TmPyPB) as an electron transporting layer (ETL). A final LiF/Al layer was then deposited to serve as a cathode. The current–voltage (I–V) characteristics of the fabricated OLEDs were measured with an experimental setup consisting of a Keithley 2400 source meter combined with a calibrated photodiode. All measurements and data acquisition were controlled by National Instrument's LabVIEW software. A spectroradiometer (Minolta, CS1000, Japan) was also employed to measure the electroluminescence spectrum of the 4 × 6 mm^2^ emitting area of the devices. The flexibility of the fabricated devices was evaluated in a glove box with the same methods used for evaluating the composite electrodes.

## Author Contributions

J.W.K. and C.J.H. designed and supervised the research. K.H.O., Y.K., C.J.L. S.J.H. and M.G.K. participated in the fabrication of the composite electrode, evaluation, and data interpretation. J.K. and S.R.P. fabricated the devices. N.K. evaluated the flexibility of the electrode. J.W.K. and Y.K. wrote the paper. All authors discussed the results and commented on the manuscript.

## Supplementary Material

Supplementary Information Supplementary information

Supplementary Information Supplementary Movie S1

Supplementary Information Supplementary Movie S2

Supplementary Information Supplementary Movie S3
