# Supplementary material for: Ultra-thin and smooth transparent electrode for flexible and leakage-free organic light-emitting diodes
Source: Sci Rep. 2015 Mar 31;5:9464. doi: 10.1038/srep09464 (PMC4379502; doi:10.1038/srep09464)
Supplement: Supplementary Information [file srep09464-s1.pdf]

Supplementary Information

Ultra-thin and smooth transparent electrode for flexible and leakage-free organic light-emitting diodes

Ki-Hun Ok,[1‡] Jiwan Kim,[2‡] So-Ra Park,[2] Youngmin Kim,[1] Chan-Jae Lee,[1] Sung-Jei Hong,[1] Min-Gi Kwak,[1] Namsu Kim,[3] Chul Jong Han,[2] Jong-Woong Kim[1*]*

[1] Display Components & Materials Research Center, Korea Electronics Technology Institute, Seongnam 463-816, Korea

[2] Display Convergence Research Center, Korea Electronics Technology Institute, Seongnam 463-816, Korea

[3] Department of Mechanical Design and Production Engineering, School of Engineering, Konkuk University, Seoul 143-701, Korea

[‡] These authors equally contributed to this work

[*] Corresponding author: Chul Jong Han (cjhan@keti.re.kr), Jong-Woong Kim (wyjd@keti.re.kr)

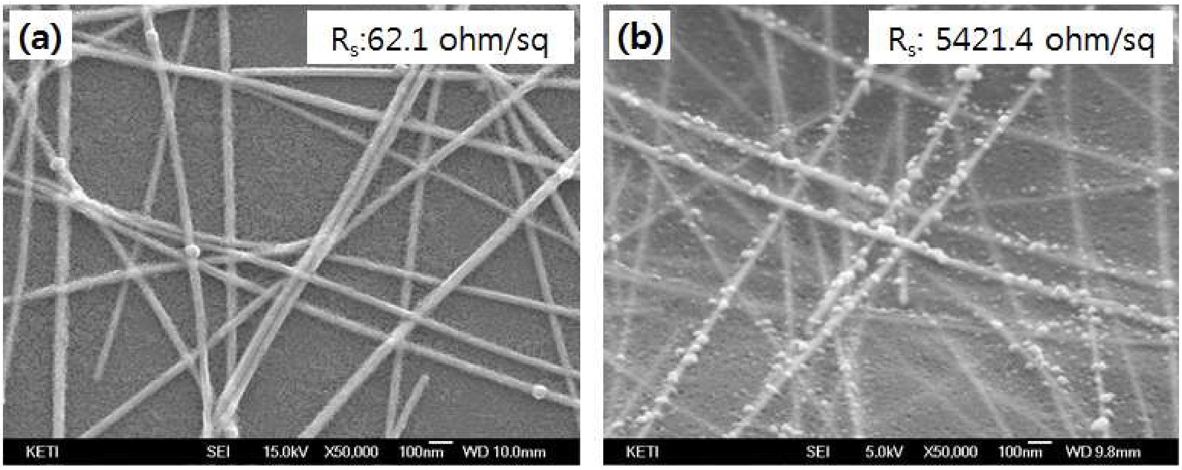

Figure S1. Reliability of PEDOT:PSS/AgNWs/glass under high temperature and humid environment: (a) an SEM micrograph for an as-prepared sample, (b) an SEM micrograph for the sample after 168 hours of storage in 85℃ and 85% relative humidity environment.

After 168 hours of a storage in high temperature and humid environment, a numerous number of particles were formed on the surface of AgNWs, and the sheet resistance of the electrode largely increased. The PEDOT:PSS used for this experiment was a commercial acidic one (Baytron P VP AI4083), and it was spin-coated on a AgNW networks formed on a glass. Before coating of PEDOT:PSS, for achieving lower sheet resistance of the AgNW networks, PVP formed on the surface of AgNWs was removed by thermal annealing at 120℃.

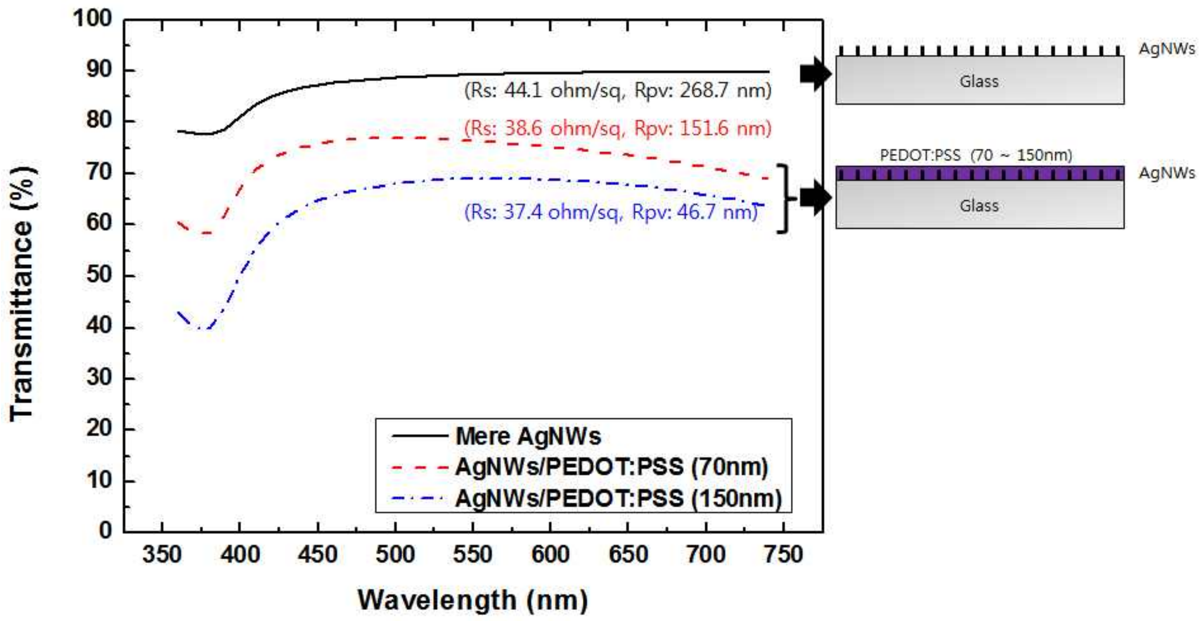

Figure S2. Effects of PEDOT:PSS coating on transmittance, sheet resistance and Rpv of the electrodes.

A coating of PEDOT:PSS on AgNW networks pre-formed on a glass was not so efficient to decrease the sheet resistance of the electrode, but somewhat effective to reduce the roughness of the electrode surface. In this case, however, it should be noted that it was at an expense of a large decrease of transmittance especially at the lower range of wavelength. Because of good conductivity of AgNWs, this effect is not a special case, but normal in most of the hybridization with other materials such as graphene, carbon nanotubes and conductive polymers.

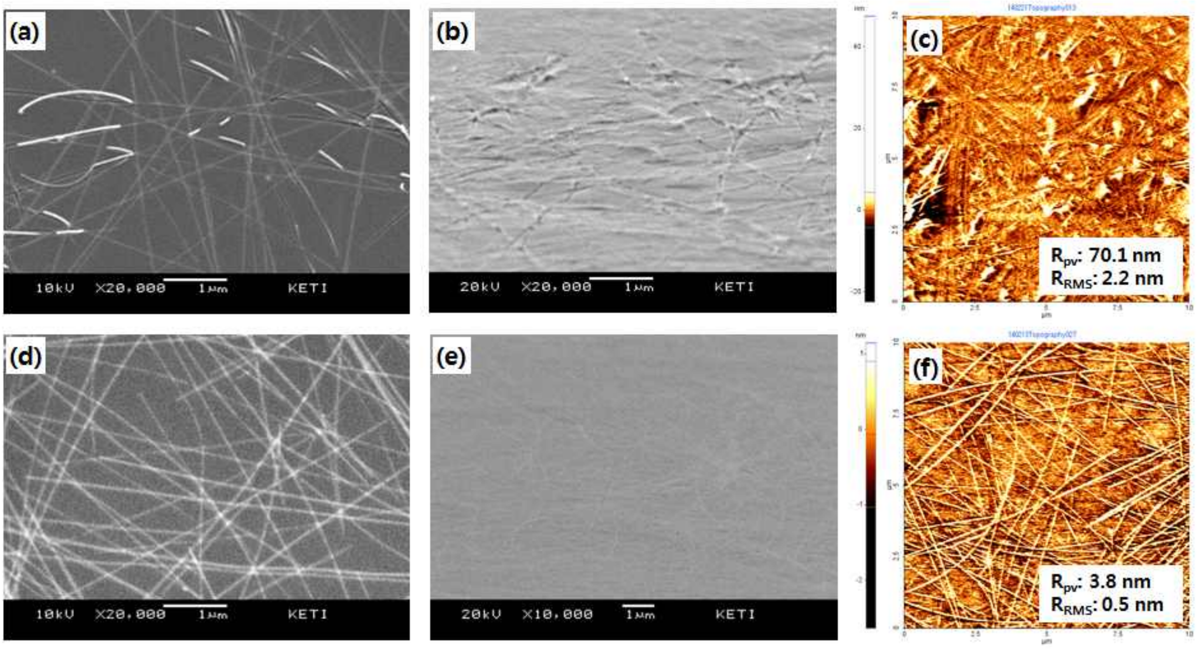

Figure S3. Effect of an employment of a sacrificial layer: (a)-(c) a sample peeled-off without a sacrificial layer, (d)-(f) a sample with a sacrificial layer, (a) and (d) top-view image, (b) and (e) 60° tilted view, (c) and (f) morphologies analyzed by AFM.

Figure S3 shows an effectiveness of a sacrificial layer employment for a better peeling-off procedure. For the AgNW-polymer composite electrode, a smooth release of a composite electrode could be realized by a larger interfacial strength between AgNWs and polymer than that between the polymer and a supporting substrate. In a case with cPI employed here, the cPI was well adhered to both AgNWs and a glass, but a little stronger to a glass. This made the nanowires sticking out of the embedded sites after peeling-off without an introduction of any sacrificing process. However, an extremely smooth surface morphology could be achieved by an employment of an efficient sacrificing procedure, as can be seen in Figure S3 (d)-(f).

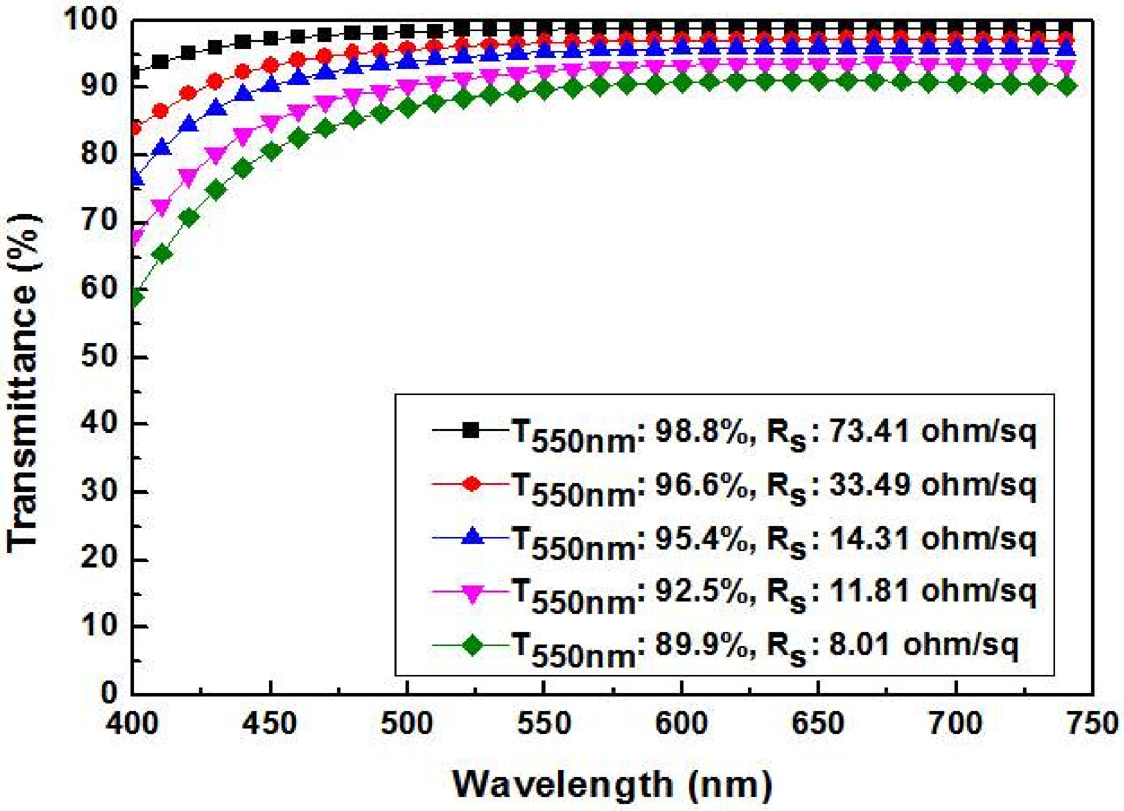

Figure S4. Measured transmittance and corresponding sheet resistance of the fabricated transparent electrodes (reference for measurements: bare cPI film).

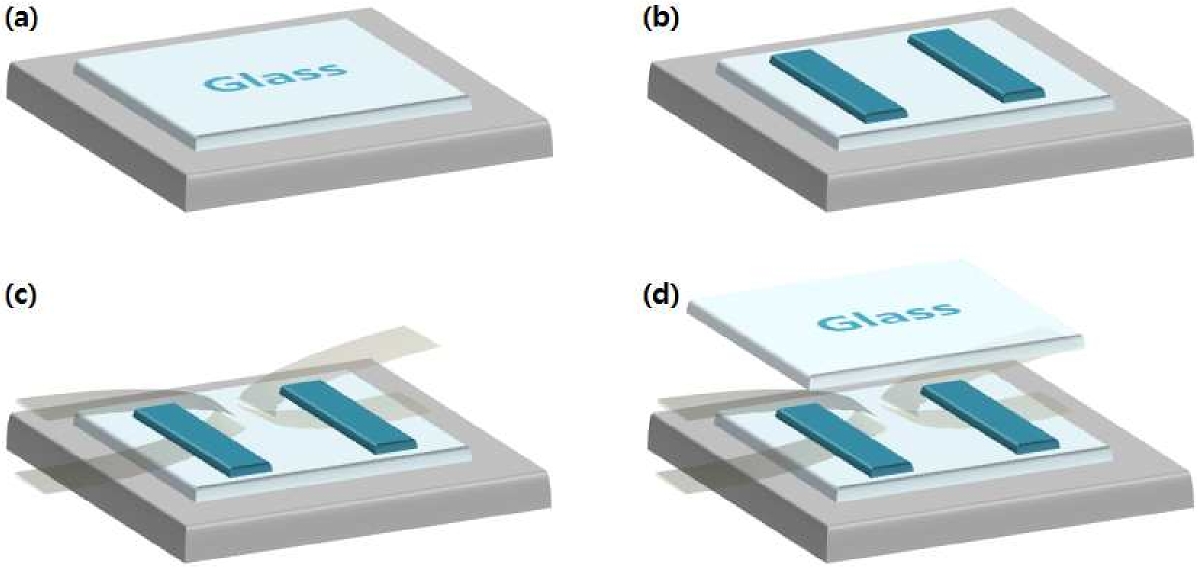

Figure S5. Schematic description of a procedure for folding test of the fabricated films: (a) placing a glass on an aluminium plate, (b) films or glasses with various thicknesses are placed on the glass, (c) placing the fabricated films for testing, (d) placing another glass on the samples.

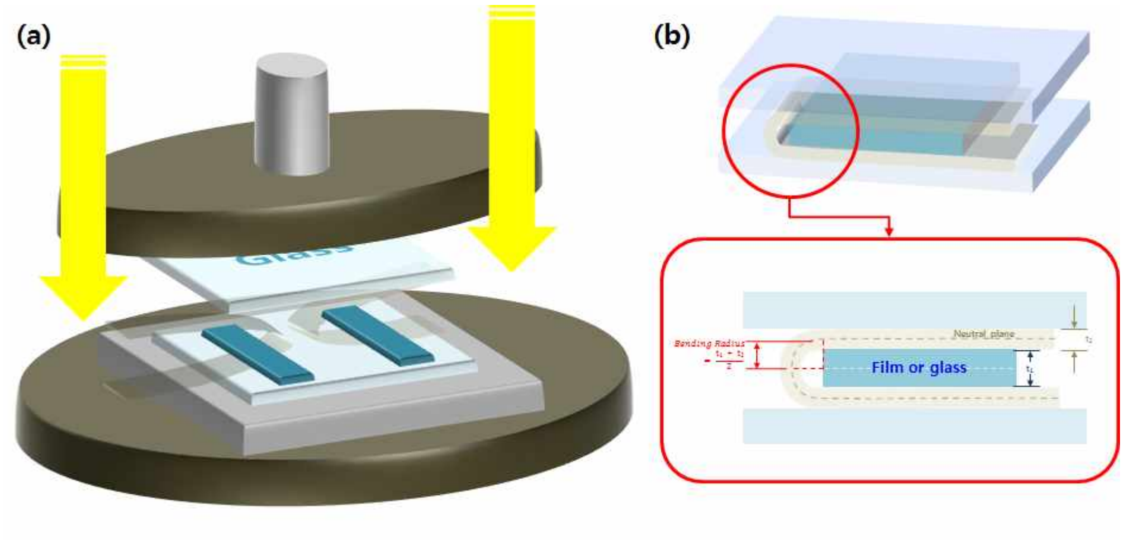

Figure S6. Schematic description of a bending test for mechanical characterization of the fabricated films: (a) a description of the test setup, (b) a detailed description of the cross-sectional view.

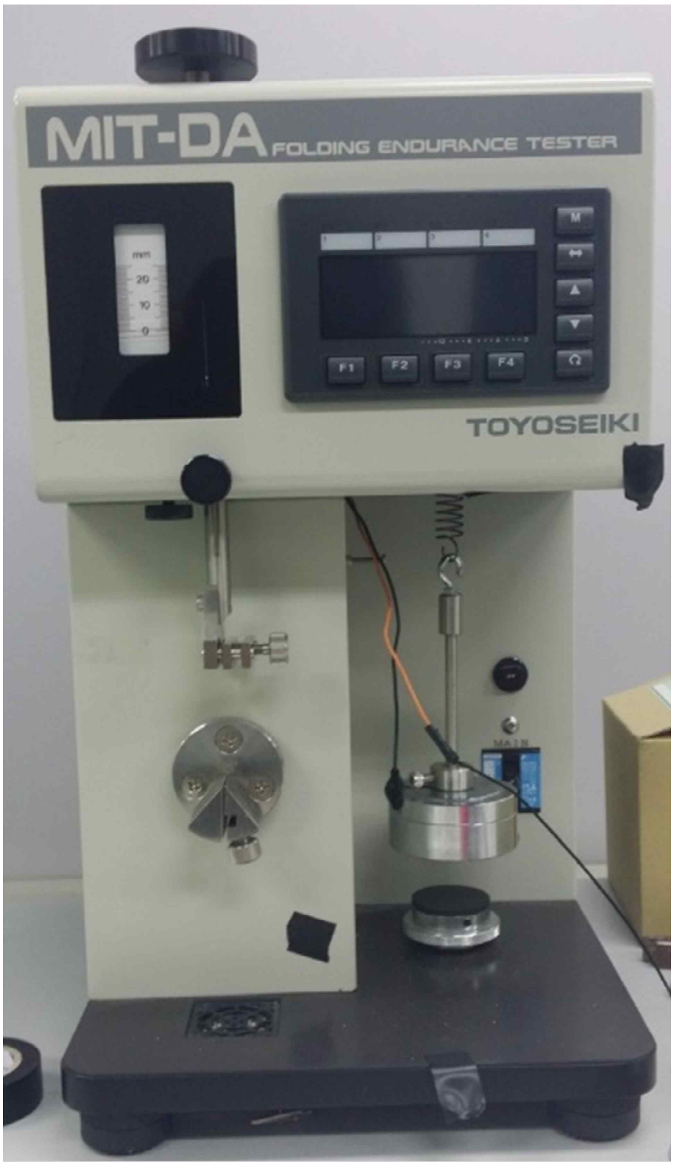

Figure S7. Bending endurance tester for evaluating cyclic bending stability.

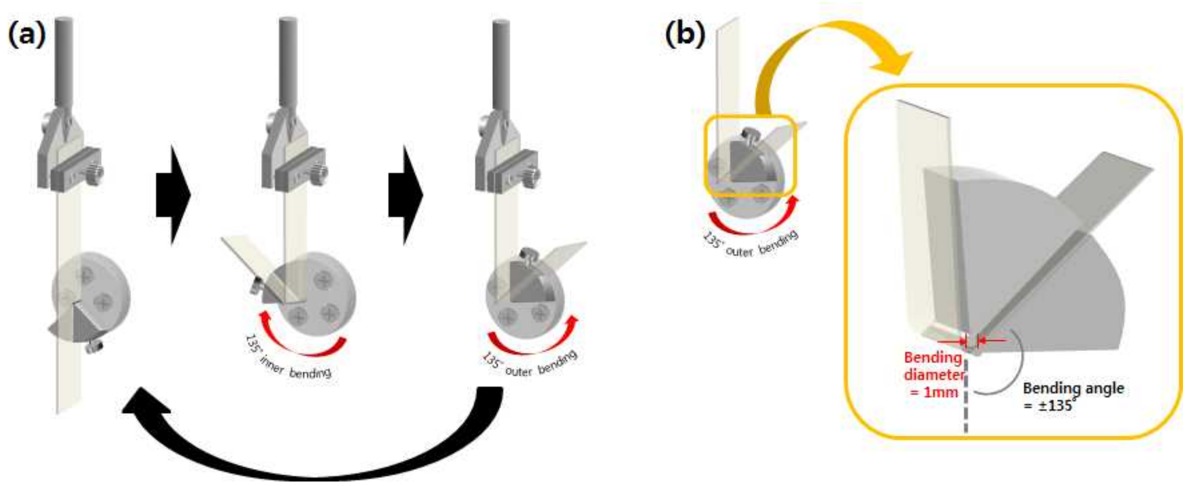

Figure S8. Schematic description of bending sequences for mechanical characterization of the fabricated samples.

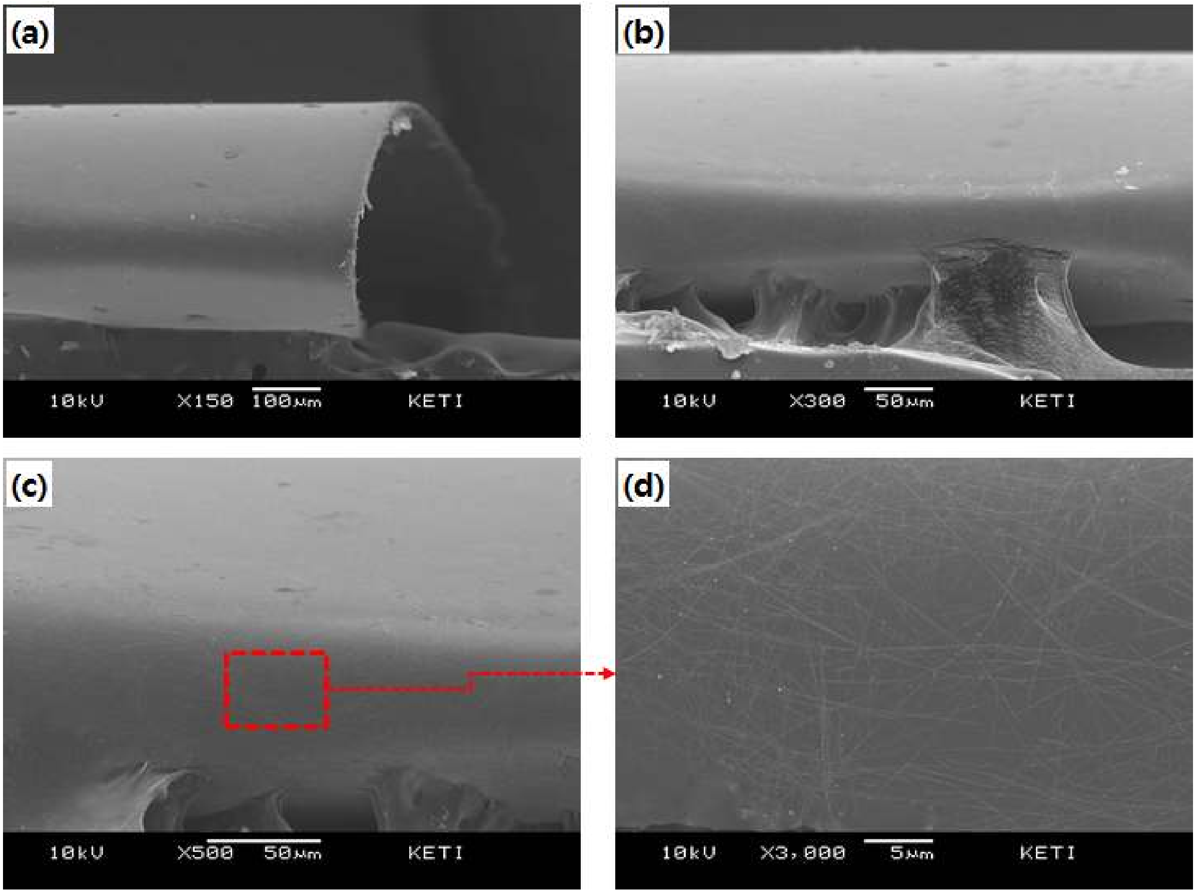

Figure S9. SEM micrographs of the surfaces for a bent sample (bending radius: 200 um): (a) overall view of the bent sample, (b) enlarged view of the center area, (c) X500 view of the center area, (d) X3000 view of the center area.

As shown in Figure S9, it could not be found that the embedded nanowires were sticking out of the composite surfaces, even after a severe bending or folding. This was mainly attributed to the fact that the adhesion between AgNWs and cPI was enough to make them resist the exfoliating forces formed during bending.

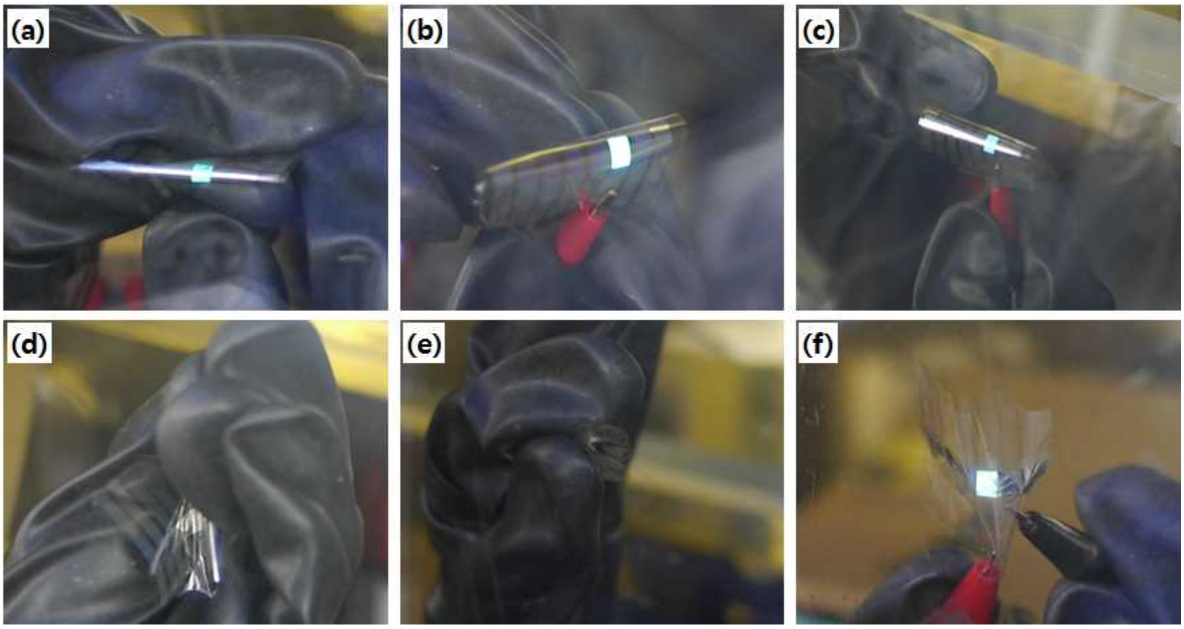

Figure S10. Flexibility of the fabricated devices: (a)-(c) lighting devices with bending, (d)-(e) crushing of the devices for testing, (f) a lighting device after an unfolding of the crushed sample.

The OLEDs fabricated on the electrode was extremely flexible. Also see the supporting movies submitted. Some dark spots on the lighting devices are seen, but they were originated from the cracks formed at the surface of Al cathode (top electrode) shown in Figure 7 (d). We could not find any of the degradation of the composite electrode after bending and folding tests.

Supplementary Movies

Supplementary Movie S1: A lighting sample with a specific curvature.

Supplementary Movie S2: A lighting sample with several bending, crushing and folding motions. Even after various stressing sequences, the lighting sample was not severely damaged.

Supplementary Movie S3: A folded lighting sample.
